# The Effect of Sunscreens on the Skin Barrier

**DOI:** 10.3390/life12122083

**Published:** 2022-12-12

**Authors:** Alicia Gonzalez-Bravo, Trinidad Montero-Vilchez, Salvador Arias-Santiago, Agustin Buendia-Eisman

**Affiliations:** 1 Dermatology Department, Faculty of Medicine, University of Granada, 18001 Granada, Spain; 2 Dermatology Department, Virgen de las Nieves University Hospital, 18012 Granada, Spain; 3 Instituto de Investigación Biosanitaria ibs.GRANADA, 18012 Granada, Spain

**Keywords:** cutaneous homeostasis, hydration, sunscreens, skin-barrier function, transepidermal water-loss

## Abstract

Transepidermal water-loss (TEWL), stratum-corneum hydration (SCH), erythema, elasticity, pH and melanin, are parameters of the epidermal barrier function and skin homeostasis that objectively indicate the integrity of the skin barrier. Sunscreens are necessary to protect people from skin cancer, but could modify the skin barrier function. Nevertheless, there are not many studies on their impact on skin homeostasis. The aim of this study is to evaluate the impact of sunscreens on the epidermal-barrier function and skin homeostasis of healthy individuals. A prospective observational study was designed. TEWL, SCH, erythema, elasticity, pH and melanin were measured on the cheek and volar region of the forearm, using non-invasive methods before and after applying sunscreen. Four different sunscreens were tested, one full-body sunscreen and three facial sunscreens. The study included 51 healthy volunteers, 72.5% (37/51) women, with a mean age of 41.63 years. After full-body sunscreen application, temperature increased by 0.68 °C (*p* < 0.001), pH by 0.16 units (*p* < 0.001), and elasticity by 0.22% (*p* = 0.039), while melanin decreased by 10.95 AU (*p* < 0.001), erythema by 28.79 AU (*p* < 0.001) and TEWL by 0.66 g·m^−2^·h^−1^ (*p* = 0.019). On the cheek, facial sunscreen 1 increased temperature by 0.51 °C, TEWL 0.7 g·m^−2^·h^−1^ (*p* < 0.05), pH by 0.12 units (*p* < 0.001) and elasticity by 0.059% (*p* < 0.001), but decreased erythema by 19.87 AU (*p* < 0.05) and SCH by 5.63 AU (*p* < 0.001). Facial sunscreen 2 increased temperature by 0.67 °C, TEWL by 1.93 g·m^−2^·h^−1^ (*p* < 0.001), pH by 0.42 units (*p* < 0.001) and elasticity by 0.12% (*p* < 0.01), but decreased melanin by 15.2 AU (*p* = 0.000), erythema by 38.61 AU (*p* < 0.05) and SCH by 10.80 AU (*p* < 0.01). Facial sunscreen 3 increased temperature by 1.15 °C, TEWL by 2.29 g·m^−2^·h^−1^ (*p* < 0.001), pH by 0.46 units (*p* < 0.001) and elasticity by 0.15% (*p* < 0.01), but decreased erythema by 35.7 (*p* < 0.05) and SCH by 10.80 AU (*p* < 0.01). In conclusion, sunscreen could slightly modify the skin-barrier function. All of them decreased erythema, likely in relation to anti-inflammatory power.

## 1. Introduction

The skin is the largest organ of the human body, and fulfills numerous defensive and regulatory functions [1]. Its general structure has three main layers, called the epidermis, dermis and hypodermis. The skin-barrier function resides mainly in the epidermis, especially in the stratum corneum [2]. The epidermal barrier maintains skin homeostasis and protects the body against numerous external factors, such as chemical, environmental and physical stress, including ultraviolet (UV) radiation. It is important to highlight the individual characteristics and behavior of the epidermal barrier, as homeostasis differs according to individual phototypes and skin-exposure behaviors [3].

It is known that solar radiation has both harmful and beneficial effects on humans, for example, ultraviolet B radiation produces erythema and DNA damage; simultaneously, it induces the synthesis of previtamin D3. In addition to UVB rays, it has been shown that UVA1 rays also induce erythema, a very important factor in the pathogenesis of melanoma and other skin tumors such as keratinocytic carcinoma [4]. However, UVA rays also contribute to blood-pressure control and cardioprotection by inducing nitric oxide release from photosensitive derivatives of intracutaneous nitric oxide.

When a new sunscreen is developed, it should provide sun protection against UVB and UVA. However, other aspects should be considered, such as the impact on skin homeostasis [5]. Sunscreen should not damage the skin, as a dysfunctional skin is more prone to be harmed by sun exposure. Moreover, a sunscren has to be cosmetically pleasing and easy to apply and spread, in order to stimulate compliance and to maintain a uniform concentration of UV filters across the skin surface, with a homogeneous film [6].

There are several parameters indicative of the integrity of the skin barrier, and among them transepidermal water-loss (TEWL) is considered one of the most important. It is defined as the flux density of water diffusing from the dermis and epidermis through the stratum corneum to the skin surface. Increased TEWL levels are associated with alterations of the skin barrier [7]. Stratum-corneum hydration (SCH) is another important parameter for assessing the barrier function of the skin. It shows the water content of the stratum corneum, and low SCH values are often associated with dermatological conditions and increased disease severity. Other skin characteristics related to skin-barrier function are pH, elasticity, temperature, melanin and the erythema index [8,9,10,11].

It is important to know how sunscreens impact on the skin barrier.

Despite its importance, there is scarce information regarding skin homeostasis after sunscreen use. Therefore, the aim of this study is to evaluate the impact of sunscreens on skin homeostasis in healthy individuals, and to assess the impact of sex and age.

## 2. Materials and Methods

### 2.1. Study Design

A prospective observational-study to evaluate the impact of sunscreens on epidermal barrier-function and skin homeostasis was designed. Participants were recruited between February 2022 and May 2022.

### 2.2. Study Population

We included healthy volunteers attending the Dermatology Department at the Hospital Universitario Virgen de las Nieves for common health conditions, such as seborrheic keratosis or melanocytic nevus, as well as students of the Faculty of Medicine of the University of Granada, with ages between 18 and 70 years. Individuals with any type of inflammatory skin disease (such as psoriasis or atopic dermatitis), those who were receiving any treatment that could alter the epidermal barrier, or who did not sign the informed consent form, were excluded.

### 2.3. Sunscreen

Four sunscreens were tested, one on the volar forearm and three on the cheek. The composition of the sunscreen is described below:

- Full-body sunscreen (applied on the volar forearm): Anthelios Spray Invisible spf50+ Broad spectrum UVA/UVB B;
- Facial sunscreen 1: Anthelios Age Correct spf50+ designed against UVB/UVA, IR-A rays, contains Fragmented Hyaluronic Acid + Phe-Resorcinol + Niacinamide;
- Facial sunscreen 2: UVMUNE40 Crema Hidratante spf50+ broad spectrum ULTRA-LONG UVA/UVA/UVB;
- Facial sunscreen 3: Hyalu B5 Aquagel spf30+, moisturizing gel containing pure Hyaluronic Acid, Vitamin B5, Vitamin E (Antioxidant Complex) and Thermal Spring Water.

### 2.4. Variables

The main variables studied were homeostasis parameters related to the epidermal barrier-function:

- Transepidermal water-loss (TEWL) in g·m^−2^·h^−1^: using the Tewameter^®^ TM 300 (open chamber) which indirectly calculates TEWL by analyzing water evaporation using diffusion principles.
- Stratum-corneum hydration (SCH), in arbitrary units (AU), using the Corneometer^®^ CM 825. It measures hydration based on the measurement of the capacitance of a dielectric medium. The probe emits an electric field that penetrates the skin and determines the dielectric constant of the water.
- Skin temperature: measured in °C, using the Skin Thermometer ST 500: the principle is based on the measurement of the infrared radiation emitted by the skin, an indicator of the skin’s microcirculation.
- Skin’s pH: measured in pH units, using the Skin-pH-Meter PH 905. This probe consists of a rod with a buffer liquid inside that acts as an electrode, allowing the identification of the potential difference between the solution inside the rod and the skin surface.
- Skin elasticity by means of the R2 value measured in %, using the Cut-ometer^®^ Dual MPA 580, based on the suction and relaxation method. The probe generates a negative pressure by suctioning the skin into an opening present in the probe, and an optical system is used to measure how much skin penetrates into it. The results evaluate the skin’s resistance to suction, i.e., firmness, and the ability to recover its original state (elasticity).
- Erythema and melanin index: in AU, using the Mexameter^®^ MX 18 by means of the MPA multiple probe adapter: it allows measurement of the two components mainly responsible for skin color: melanin and hemoglobin (erythema). The process is based on a light absorption/reflection principle, using a sensor that emits light at three specific wavelengths, and the receiver measures the light reflected by the skin. By defining the amount of light that is emitted, it is possible to calculate the amount of light that has been absorbed by the skin.

Measurements were carried out using all these probes (Tewameter^®^ TM 300, Corneometer^®^ CM825, pHmeter^®^ PH905, Mexameter^®^ MX18, Cut-ometer^®^ Dual MPA 580) adapted to an MPA 580 multiprobe system (MPA COURAGE+KHAZAKA electronicGmbH, MICROCAYA, S.L, Bilbo, Spain).

Full-body sunscreen was tested on the volar region of the right forearm, and facial sunscreens were used on the left cheek two centimeters from the external canthus of the eye. The volar forearm was divided into two areas (a region without sunscreen—the control area—and an area where the sunscreen was applied). The cheek was divided into 4 areas (an area without sunscreen—the control area—and areas 1, 2, and 3, where each facial sunscreen was applied). The sunscreen was applied to each area and measures were taken after 20 min of application. All variables were also measured on the control area before (basal measure) and 20 min after (control measure).

The same amount of each sunscreen (0.05 mg) was applied to each area. The measurements of the different parameters were performed under the same conditions of humidity (40–50% relative) and temperature (23 ± 1 °C). Likewise, the participants were instructed to suspend any type of skin care from the previous night, and not to use makeup on the day of the test. Prior to the measurement, participants were given an acclimatization time of 5–10 min. Measurements were performed with the patients in supine position on a couch.

Secondary variables were gathered in a clinical interview including participants’ sociodemographic characteristics that could influence epidermal barrier-function: sex, age, phototype, occupation, toxic habits, skin hydration, use of topical corticosteroids, sun exposure, use of sunscreens, personal history of inflammatory skin-disease, concomitant diseases, current medication intake and some anthropometric measurements, such as weight (kg), height (m) or body mass index (BMI). (APPENDIX II: data collection sheet). To assess the impact of sex and age, the population was divided into men and women and into participants <40 and ≥40 years old.

### 2.5. Statistical Analysis

Categorical variables were expressed as relative (absolute frequencies), and continuous variables as the mean (standard deviation). The Kolmogorov–Smirnov test was used to check the normality of the data distribution. Categorical data was compared using the chi-square test. Continuous independent variables were contrasted using Student’s *t*-test for independent variables. To compare the homeostasis parameters before and after application of the sunscreen, Student’s *t*-test for paired samples was used. A *p*-value of <0.05 is considered statistically significant. Statistical analyses were performed with the SPSS package (SPSS for Windows, version 24.0 Chicago: SPSS Inc., Chicago, IL, USA).

## 3. Results

Fifty-one healthy volunteers were included, 37 women (72.5%) and 14 men (27.55%). The mean age of the sample was 41.63 years. The rest of the characteristics of the sample are shown and detailed in Table 1.

### 3.1. Changes in Skin-Barrier Function with a Full-Body Sunscreen

**Figure 1:**
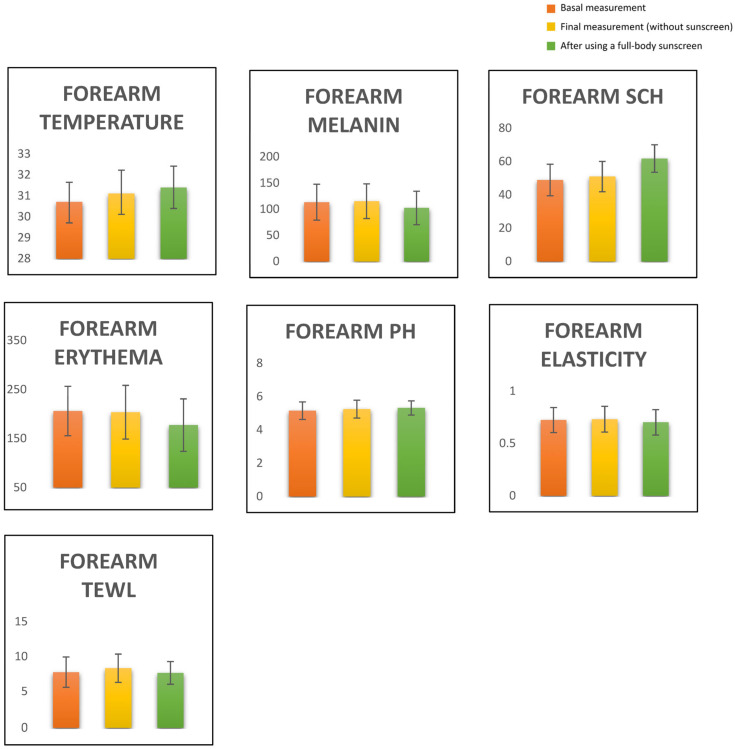
Changes in skin-barrier function with a full-body sunscreen.

Changes in skin-barrier-function parameters after using a full-body sunscreen are shown in Figure 1, Table 2. There was no change comparing basal measures with the final control measure on the volar forearm. After using the full-body sunscreen, most parameters changed slightly, except TEWL. An increase in temperature of 0.69 °C (*p* < 0.001), a decrease in melanin of 10.95 AU (*p* = 0.000), a decrease in erythema of 28.79 AU (*p* < 0.001), an increase in pH of 0.16 (*p* = 0.006), an increase in SCH of 12.91 AU (*p* < 0.001) and a decrease in elasticity of 0.022% (*p* = 0.039) were observed.

### 3.2. Changes in Skin-Barrier Function with Facial Sunscreens

**Figure 2:**
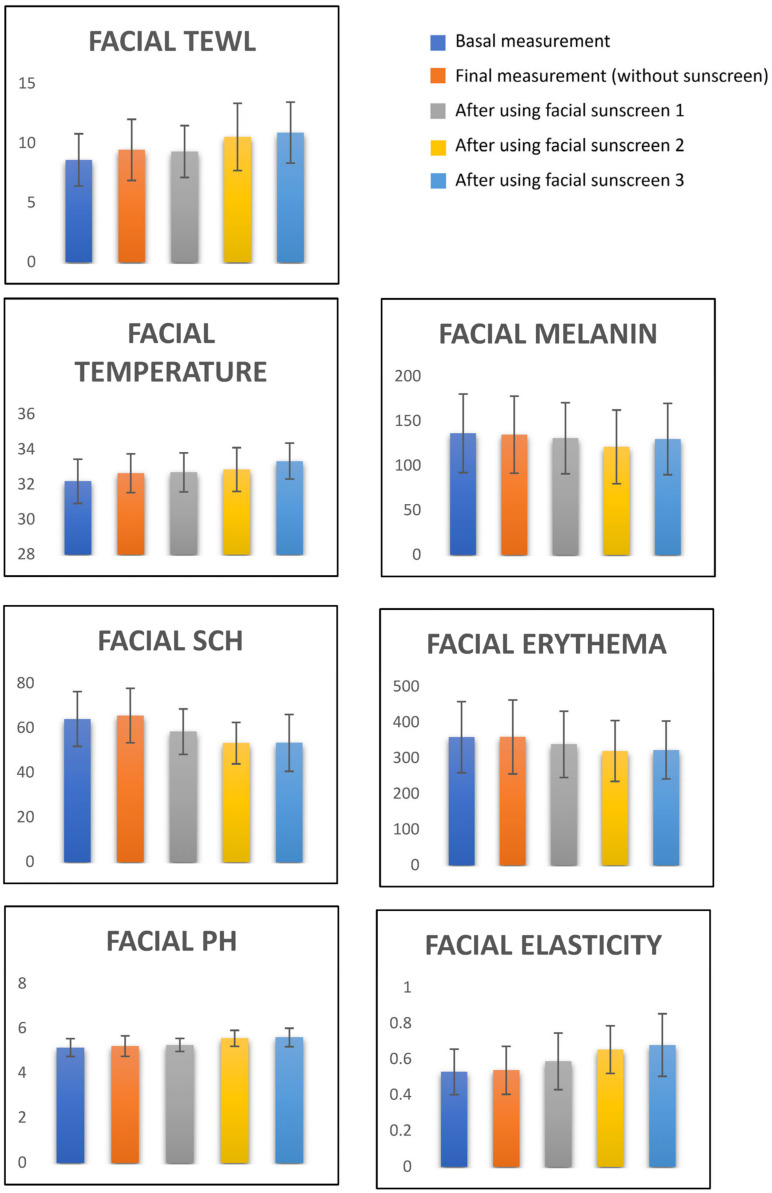
Changes in skin barrier-function with three facial sunscreens.

Changes in skin-barrier-function parameters after using three facial sunscreens are shown in Table 2, Figure 2. There was no change comparing basal measures with the final control-measure on the volar forearm. Changes were observed after all sunscreen applications. After the application of facial sunscreen 1, an increase in temperature of 0.51 °C (*p* < 0.001), a decrease in facial erythema of 19.87 AU (*p* = 0.009), an increase in pH of 0. 12 (*p* = 0.016), an increase in TEWL of 0.71 g·m^−2^·h^−1^ (*p* = 0.039), a decrease in SCH of 5.64 AU (*p* = 0.009), and an increase in elasticity of 0.059% (*p* = 0.006), were observed. After application of sunscreen 2, a temperature increase of 0.67 °C (*p* < 0.001), a decrease in melanin of 15.26 units (*p* < 0.001), a decrease in facial erythema of 38.61 AU (*p* < 0.001), an increase in pH of 0.42 (*p* < 0.001), an increase in TEWL of 1.93 g·m^−2^·h^−1^ (*p* < 0.001), a decrease in SCH of 10.81 AU (*p* < 0.001) and an increase in elasticity of 0.1246% (*p* < 0.001), were observed. After application of sunscreen 3 to the cheek, a temperature increase of 1.15 °C (*p* < 0.001), a decrease in facial erythema of 35.71 units (*p* < 0.001), an increase in pH of 0.46 (*p* < 0.001), an increase in TEWL of 2.29 g·m^−2^·h^−1^ (*p* < 0.001), a decrease in SCH of 10.67 AU (*p* < 0.001) and an increase in elasticity of 0.1502% (*p* < 0.001), were observed. The facial sunscreen that increased temperature, pH, TEWL and elasticity the most was cream 3, while cream 2 decreased melanin, erythema and hydration the most.

### 3.3. The Impact of Sex on Skin-Barrier Function Using Sunscreens

Regarding full-body sunscreen, changes between basal and final control-measures were similar for men and women. After using the full-body sunscreen, pH (*p* = 0.005) and SCH (*p* = 0.012) decreased only in women and TEWL decreased only in men (*p* = 0.039), as shown in Table 3.

Concerning facial sunscreens, changes between basal and final control-measures were similar for men and women. After applying the first sunscreen, changes in SCH were different, as it decreased in men and increased in women (*p* = 0.048). No difference in changes were observed between men and women after applying facial sunscreen 2 and 3, (Table 4).

### 3.4. The Impact of Age on Skin-Barrier Function Using Sunscreens

Regarding full-body sunscreen, changes between basal and final control-measures were similar for participants <40 and ≥40 years old, except for pH (Table 5). After applying the full-body sunscreen, it was observed that the erythema increase was greater in patients ≥40 (+16.85 vs. +39.41, *p* = 0.027).

Concerning facial sunscreens, changes between basal and final control-measures were similar for participants <40 and ≥40 years old, except for elasticity. We only found significant differences in the elasticity parameter after applying sunscreen 3. The decrease in elasticity was higher in participants <40 (−0.088 vs. −0.196, *p* = 0.018) (Table 6).

## 4. Discussion

The results obtained in our study shows that there are differences after applying sunscreen. Nevertheless, these differences were slight, and some of these parameters even improved.

There is scarce literature on the use of sunscreens and their effect on skin homeostasis. In fact, no previous research study has been performed comparing skin-homeostasis parameters before and after the application of a sunscreen. Although there have been studies on TEWL and temperature after the use of sunscreens, these were oriented to the use of sunscreens during physical exercise [8,9].

As for temperature, our study shows a rise in temperature after the use of sunscreens, with an average increase of 0.76 °C on the face and 0.68 °C on the forearm. These results are contrary to those obtained by Ou-Yang et al. in their study regarding the impact of sunscreens on the skin during exercise, as they did not observe any change [8]. However, it is important to highlight the fact that the method used to obtain the measurements was different for the two studies. Ou-Yang took the first temperature immediately after applying the sunscreen to people doing physical exercise [8], and we evaluated changes after 20 min in resting conditions, without including physical exercise. Despite discrepancies, the increase in temperature experienced in our study is not significant, and remains within the normal parameters of skin temperature established by Benedict et al. In their work, they determine a normal range between 31 and 35 °C of temperature, depending on the body zone, with a higher temperature in the facial zone and lower in more distal zones such as the forearm [9,10]. This distribution is also present in our data, in which we observe that the facial temperature is higher than the one obtained on the forearm and that both are within the normal range.

Both melanin and erythema experience a decrease after the application of sunscreen, and although melanin is not one of the most important parameters for indicating the integrity of the epidermal barrier, erythema is a good indicator of the action of irritants on it, as it is a cardinal sign of inflammation [11,12,13]. In our study, erythema decreased considerably with respect to skin without sunscreens, so we can determine that, in addition to not irritating the skin, they improve this parameter. In addition, since erythema is a cardinal sign of inflammation and its reduction occurs with all sunscreens, there is a possibility that these have an anti-inflammatory effect, so the study of sunscreens could be expanded in future research projects, since there is no literature on this subject. Nevertheless, it is also possible that sunscreen use alters the colorimeter measurements.

Skin pH is another essential parameter for the evaluation of epidermal functions, as the acidic nature of pH influences skin-barrier function, lipid synthesis and aggregation, epidermal differentiation, desquamation, skin-barrier regeneration and skin antimicrobial-response [14,15]. Elevated pH values are related to the loss of antimicrobial activity, and it has also been shown that, in patients with atopic dermatitis, higher values of the SCORAD index are associated with skin-barrier dysfunction, which is reflected in higher pH and temperature and lower SCH and elasticity [15]. In our study we observed that despite the increase in pH after the application of the sunscreen, the values remain within normal ranges, and therefore the epidermal barrier is not affected by it.

TEWL is one of the most important characteristics of the skin barrier, and numerous studies have shown that high TEWL values are often associated with skin-barrier deficiencies, and lower TEWL with healthy skin [16,17,18,19,20]. There is also evidence that TEWL decreases with age, which could be misinterpreted as an improvement in the skin barrier [17,21]. TEWL is influenced by many environmental and individual factors, such as age, sex, race, anatomical location, skin temperature and other environmental conditions such as season, smoking habits, type of measurement-technique used, and many other factors [22,23,24]. The normal range of TEWL is 1 to 25 g/m²/h, and, as we have previously stated, values above this limit indicate dysfunction of the epidermal barrier [20,25] They also follow a different distribution in terms of location, so that it appears to be greater in the facial area compared to other parts of the body such as the forearm [17]. In our case, we obtained different results depending on the area, with an improvement of the parameter in the forearm but a slight increase in the facial area. Despite this, they results remain in the normal range.

SCH is another important parameter for skin integrity, and lower-than-normal values are also frequently associated with skin-barrier dysfunction. In some publications it has been observed that the face has higher hydration-indices than other anatomical regions [26,27,28]; however, there is also literature in which the measurements are reversed, and the skin of the forearm is more hydrated than that of the face [27]. In our case, our findings were consistent with increased facial-hydration.

Elasticity is another important characteristic related to the biomechanical properties of the skin. A decrease in elasticity has been related to a higher SCORAD index in patients with atopic dermatitis, and has been observed to be affected at older ages, decreasing as age increases [16]. In our case, facial elasticity increased after the application of sunscreens, especially with facial sunscreen 3, which could be explained by the presence of antioxidants and especially by the presence of pure hyaluronic acid in its formula, an active ingredient known for its viscoelastic properties and one of the main components of the extracellular matrix [29,30].

The variations between the sunscreens could be due to differences in their composition but also to the differences between the two anatomical regions. It has been shown that the skin of the face is thinner than that of the rest of the body, and that its stratum corneum has fewer layers of corneocytes [10,25], so the effect that the different components have could be greater at this level. In addition, several studies have shown that water-based emollients increase TEWL in psoriasis patients [6], which could explain our increase in facial TEWL, as they are water-based sunscreens.

We chose sunscreens with different compositions and different vehicle formulations. Further research could be carried out to assess whether the same composition in a different vehicle could modify skin-barrier function in a different way. It could be also interesting to develop research to assess if changes in only one excipient may alter the impact of the sunscreen on skin-barrier function.

Concerning the impact of sex and age, we did not observe great differences between men and women or participants of different age-groups. This fact is important so that recommendations about sunscreen regarding skin-barrier function could be spread to both sexes and different age-groups. It is important to mention that we only include adults, so further research is needed to evaluate the impact of sunscreen on children.

Our study is subject to several limitations. The sample size could be enlarged, to increase the significance of the data obtained. In addition, the sample may not be representative regarding genders as the percentage of female volunteers was higher, likely in relation to an over-representation of the female sex in the Faculty of Medicine, and because women are frequently more worried about their health and are more prone to participate in investigations into creams. Finally, we also take into account the limitations mentioned by Nedelec et al. in their study regarding melanin and erythema, in that since skin characteristics are affected by seasonal variation (and measurements were performed in winter), these parameters could increase in summer, and hence not be representative

## 5. Conclusions

Sunscreen could slightly modify skin-barrier function.

Erythema and elasticity improved with respect to baseline measurements, especially erythema, which decreased significantly with the use of all sunscreens. On the other hand, pH, TEWL and SCH decreased slightly. However, despite these changes, they are still within the normal range.

We consider that sunscreens ultimately do not significantly alter skin homeostasis and that their benefits regarding skin-cancer prevention outweigh epidermal-barrier modifications. For this reason, we consider that their use is fundamental.

Finally, we would like to emphasize that this work may serve as a starting point for future studies on the possible anti-inflammatory effect of sunscreens, an effect that has not been investigated so far, but from which promising results could be obtained.

## Figures and Tables

**Table 1:** Sociodemographic characteristics of the sample.

Variables	Participants (N = 51)
Age	41.63 (16.45 SD)
Sex Female Male	37 (72.5%) 14 (27.5%)
Phototype I II III IV	4 (7.8%) 27 (52.9%) 18 (35.3%) 2 (3.9%)
Smoking habit (YES)	12 (23.5%)
Alcohol habit (YES)	2 (3.9%)
Mosturizing (0–7 times/week) 0 1 2 3 4 7	16 (31.4%) 1 (2%) 3 (5.9%) 2 (3.9%) 24 (47.1%)
Solar exposure (hours/week) ≥10 <10	21 (41.17%) 30 (58.83%)
Use of photoprotectors never sometimes always	13 (25.5%) 24 (47.1%) 14(27.5%)

**Table 2:** Skin-barrier-function parameters at baseline and after using the sunscreens.

Homeostasis Parameters	Initial Forearm Measurement	Final Forearm Measurement (without Cream)	*p*	Forearm with Cream	*p*	Inicial Cheek Measurement	Final Cheek Measurement (without Cream)	*p*	Cheek with Cream 1	*p*	Cheek with Cream 2	*p*	Cheek with Cream 3	*p*
Temperature (°C)	30.71	31.11	0.050	31.39	0.000	32.18	32.24	0.074	32.79	0.000	32.85	0.000	33.32	0.000
Melanin (AU)	113.23	115.22	0.167	102.28	0.000	136.11	134.59	0.458	130.59	0.088	120.85	0.000	129.68	0.065
Erythema (AU)	206.18	203.75	0.606	177.39	0.000	357.74	358.46	0.899	337.87	0.009	319.13	0.000	322.03	0.000
pH	5.15	5.25	0.050	5.32	0.006	5.14	5.21	0.074	5.26	0.016	5.56	0.000	5.59	0.000
TEWL (g·m^−2^·h^−1^)	7.76	8.32	0.069	7.66	0.724	8.59	9.43	0.039	9.39	0.039	10.52	0.000	10.88	0.000
SCH (AU)	48.91	50.99	0.056	61.81	0.000	63.99	65.57	0.050	58.36	0.009	53.19	0.000	53.33	0.000
Elasticity (%)	0.723	0.731	0.504	0.701	0.039	0.528	0.537	0.596	0.587	0.006	0.6522	0.000	0.6778	0.000

*p* value after using Student’s *t* test for paired samples. Differences between initial and final baseline controls (without photoprotection). AU = arbitrary units, TEWL = transepidermal water-loss, SCH = stratum-corneum hydration.

**Table 3:** Changes in skin-barrier function after using the full-body sunscreen, depending on the sex.

Homeostasis Parameters	Forearm Increase without Cream in Men	Forearm Increase without Cream in Women	*p*	Forearm Increase with Cream in Men	Forearm Increase with Cream in Women	*p*
Temperature (°C)	−0.57	−0.34	0.286	−0.65	−0.70	0.826
Melanin (AU)	+0.34	−2.86	0.319	+13.33	+10.04	0.531
Erythema (AU)	+1.77	+2.68	0.735	+41.30	+24.06	0.135
pH	−0.16	−0.073	0.979	−0.42	−0.07	0.005
TEWL (g·m^−2^·h^−1^)	−0.78	−0.48	0.775	−0.86	+0.46	0.039
SCH (AU)	−3.37	−1.60	0.922	−18.49	−10.79	0.012
Elasticity (%)	−0.017	−0.005	0.567	−0.004	+0.031	0.125

*p* value after using Student´s *t* test for independent samples. Differences in the increase between men and women in forearm measurements without and with sunscreen.

**Table 4:** Changes in skin-barrier function after using three facial sunscreens, depending on the sex.

Homeostasis Parameters	Face Increase without Cream in Men	Face Increase without Cream in Women	*p*	Face Increase with Cream 1 in Men	Face Increase with Cream 1 in Women	*p*	Face Increase with Cream 2 in Men	Face Increase with Cream 2 in Women	*p*	Face Increase with Cream 3 in Men	Face Increase with Cream 3 in Women	*p*
Temperature (°C)	−0.43	−0.48	0.844	−0.47	−0.53	0.833	−0.7	−0.66	0.880	−0.89	−1.24	0.232
Melanin (AU)	+5.94	−0.16	0.182	+13.05	+2.7	0.146	+14.51	+15.55	0.890	+13.27	+3.84	0.219
Erythema (AU)	−9.41	+2.58	0.344	+31.75	+15.38	0.320	+43.11	+36.91	0.736	+56.01	+28.04	0.194
pH	−0.08	−0.06	0.882	−0.26	−0.07	0.084	−0.49	−0.39	0.387	−0.51	−0.51	0.645
TEWL (g·m^−2^·h^−1^)	−1.53	−0.59	0.096	−1.05	−0.58	0.530	−1.88	−1.95	0.930	−2.28	−2.3	0.978
SCH (AU)	−2.85	−1.08	0.315	−0.97	+8.14	0.048	+5.81	+12.70	0.167	+11.75	+10.28	0.815
Elasticity (%)	−0.046	+0.005	0.192	−0.079	−0.043	0.474	−0.129	−0.124	0.913	−0.15	−0.143	0.906

*p* value after using Student´s *t* test for independent samples. Differences in the increase between men and women in forearm measurements with and without sunscreen.

**Table 5:** Changes in skin-barrier function after using the full-body sunscreen, depending on age.

Homeostasis Parameters	Forearm Increase without Cream in People ≥40 Years Old	Forearm Increase without Cream in People <40 Years Old	*p*	Forearm Increase with Cream in People ≥40 Years Old	Forearm Increase with Cream in People <40 Years Old	*p*
Temperature (°C)	−0.35	−0.46	0.572	−0.69	−0.6778	0.929
Melanin (AU)	+0.01	−3.75	0.189	+10.32	+11.50	0.802
Erythema (AU)	−0.57	+5.09	0.552	+16.85	+39.41	0.027
pH	+0.028	−0.21	0.005	−0.06	−0.26	0.076
TEWL (g·m^−2^·h^−1^)	−0.67	−0.46	0.739	+0.06	+0.14	0.888
SCH (AU)	−1.91	−2.24	0.870	−11.33	−14.30	0.292
Elasticity (%)	+0.015	−0.09	0.521	+0.031	+0.013	0.367

*p* value after using Student´s *t* test for independent samples. Differences in the increase between men and women in forearm measurements with and without sunscreen.

**Table 6:** Changes in skin-barrier function after using three facial sunscreens, depending on age.

Homeostasis Parameters	Face Increase without Cream in People ≥40 Years Old	Face Increase without Cream in People <40 Years Old	*p*	Face Increase with Cream 1 in People ≥40 Years Old	Face Increase with Cream 1 in People <40 Years Old	*p*	Face Increase with Cream 2 in People ≥40 Years Old	Face Increase with Cream 2 in People <40 Years Old	*p*	Face Increase with Cream 3 in People ≥40 Years Old	Face Increase with Cream 2 in People <40 Years Old	*p*
Temperature (°C)	−0.51	−0.42	0.687	−0.44	−0.58	0.551	−0.58	−0.7519	0.472	−1.04	−1.24	0.436
Melanin (AU)	+1.67	+1.38	0.944	+7.73	3.6	0.518	+16.49	+14.17	0.729	+8.58	+4.51	0.556
Erythema (AU)	+2.62	−3.67	0.579	+13.69	25.38	0.427	+39.89	+37.48	0.883	+20.88	+48.89	0.145
pH	−0.014	−0.12	0.176	−0.10	−0.13	0.769	−0.40	−0.44	0.712	−0.42	−0.5	0.502
TEWL (g·m^−2^·h^−1^)	−0.66	−1.01	0.493	−0.75	−0.7	0.893	−1.92	−1.94	0.987	−2.52	−2.09	0.544
SCH (AU)	−0.99	−2.08	0.499	+5.18	6.04	0.838	+9.55	+11.93	0.597	+5.89	+14.94	0.104
Elasticity (%)	+0.03	−0.040	0.043	−0.0167	−0.09	0.117	−0.10	−0.14	0.332	−0.088	−0.196	0.018

*p* value after using Student´s *t* test for independent samples. Differences in the increase between men and women in forearm measurements without and with sunscreen.

## Data Availability

The data presented in this study are available from the corresponding author on request.
